# Midori de Habich: the economist who ran Peru’s health ministry

**DOI:** 10.2471/BLT.20.030220

**Published:** 2020-02-01

## Abstract

Midori de Habich speaks to Gary Humphreys about Peru’s journey towards universal health coverage and the advantages of being an economist in a world of doctors.

Q: You studied economics at university and got your first job at the Central Bank of Peru in 1984. How did you get from there to running the country’s health ministry?

A: It’s true that my first job was at the central bank, but from the beginning the social sector was the focus of my work, and that included provision of and access to health services. The president of the bank had set up a department, which essentially looked at different social sectors, through an economic lens. This was in the mid-1980s when the bank was trying to get to grips with the aftermath of a structural adjustment plan that had been introduced by the government to deal with the country’s economic situation. We were generating poverty maps, trying to measure inequality within the country, and developing analyses based on that data.

Q: What did your work reveal?

A: We could see that the social sectors were really suffering as a result of government spending cuts. This included the health sector, where public health facilities were struggling financially and charging user fees to fund themselves. As a result, significant barriers to accessing public health services were starting to emerge.

Q: Do central banks usually get involved in analyses of the social sector?

A: No, and the work was innovative for the time. The central bank is basically there to develop and implement monetary policy, and in 1991 I was moved into the mainstream of the bank’s core functions working on macroeconomic indicators, work I found very interesting. But, in 1995, when I was invited to join a team advising the Ministry of Health as part of a USAID-funded project I accepted.

Q: What were you advising the ministry on?

A: I was asked to develop the financing and management components of the project, which was aimed at reforming the health sector.

Q: That sounds a long way from macroeconomic policy.

A: It was, the main difference being that with macroeconomic policy there is basically very little implementation. You enact policy, raising interest rates for example, and the market follows. It is completely different in the social sector, where things start to get difficult after enactment and there are lots of factors at play, and lots of stakeholders. I remember when I was first asked to work on the project I was given four years to deliver. I said, ‘Oh, I'll do it in two’ (laughing). Well 20 years later they are still struggling to implement reforms. But there has been progress, especially on the health insurance side.

“The ability to make the case for investing in health is of vital importance.”

Q: Can you say more about that?

A: When I started working with the health ministry in 1995 only about a quarter of the population had health insurance and most of them were in the formal sector, and had access to social security. At that time the government was spending about US$100 per capita on health. The picture started to change with the setting up of the *Seguro Escolar Gratuito* (SEG, Free School Insurance) and *Seguro Materno Infantil* (SMI, Maternal and Child Insurance) schemes in 1997, both schemes financed out of general tax revenues and extended health insurance to groups not covered by social security. Then, in 2002, the government merged the two schemes into the *Seguro Integral de Salud* (SIS, Comprehensive Health Insurance) scheme, which extended coverage mainly to the poor. In 2009, Peru’s Universal Health Coverage Act was passed establishing a mandatory health insurance system, which includes a *Plan Esencial de Aseguramiento en Salud* (PEAS, Essential Health Benefit Package) financed by the three pre-existing health insurance schemes. This system is intended to cover the entire population of Peru.

Q: And does it cover the whole population?

A: Not yet, but we are getting closer. Today 87% (approximately 28 of 32 million) of Peruvians have health insurance coverage. But there are still significant gaps across the health system and further reform is needed for health service delivery and health financing systems. The government is now spending around US$ 350 per capita, which is still not enough, but an improvement on 1990 levels.

Q: What was required to bring about that change?

A: Simply put, the establishment of a broad consensus around the need for change, a consensus that came out of the transition towards representative democracy after 2002. Achieving that consensus was a complex process requiring agreement and compromise between 18 political parties, and significant inputs from Peru’s academic institutions and development partners. The Ministry of Health played a key role in responding to the different stakeholders and eventually arriving at the 2009 legislation that received broad support.

Q: You were health minister from 2012 to 2014. How challenging was that, given that you came from a background in economics?

A: Very. Of course, I’d had time to familiarize myself with the main issues during my time as a consultant to the ministry. Also, I think because I came at it from an overall systems financing perspective I was well placed to fulfil my role. Ministers of health tend to come from a medical background, which does not necessarily prepare them to deal with the financial aspects of a complex system like the health sector. It's challenging for people with medical backgrounds to have a productive dialogue with the finance ministry, where important resource allocation decisions are taken after all. 

The ability to make the case for investing in health is of vital importance, especially given the tendency to deprioritize health as a “non-productive” sector compared to, for example, manufacturing or infrastructure. Also, because the return on investment in the health sector as a whole is not easily measured and may only become apparent over time, politicians tend to see health as a black hole offering little that they can hold up to the public come election time.

Q: How did you make the case for health?

A: We tried to link our funding requests to specific results. For example, we said we need this amount of money for a specific set of services that we can account for and that will have been provided at the end of a given period. We tended to have that kind of discussion rather than proposing an increase in salaries, which, of course, would also have had a significant impact on the health system, but which would have been difficult to link to specific outputs. So even though the broader objective might be to achieve greater access to medicines, we would ask for specific funding for cold chain infrastructure. Because we focused on specific deliverables, we could also be very clear about the consequences of not committing the resources needed.

“Ensuring that the focus of reform is on the needs of the population is the biggest challenge of all.”

Q: Can you elaborate?

A: For example, if the finance ministry said we don't have money for that, we’d ask exactly how much they were ready to commit and then say that with that their proposed level of funding, we would achieve the government’s objectives in 20 years. Let us take that to the president and see what he says. When we did go to the president we tended to get more money than the finance minister was initially ready to commit. Of course, in some cases it was necessary to take a longer view and understand that needed reforms cannot always be achieved within one electoral cycle. This presents its own challenges, notably with regard to stability of funding. 

Essentially, as health minister, you are making long-term plans with a financing mechanism that is short term and volatile. Finance ministers don’t like to make long-term commitments because they require discretionary power to adjust fiscal policy in response to external and internal shocks to the economy. We managed to negotiate long-term commitments, especially for major reforms, making the argument for counter cyclical funding.

Q: Can you explain that for the non-economists?

A: Counter cyclical funding means spending less and saving during periods of economic growth and spending more during periods of economic contraction. There is a very strong link between sound fiscal policy and health system funding in that both need to be sustained through the ups and downs of boom and bust. It’s an eminently reasonable approach to fiscal and health system funding, but not one that politicians always espouse. Fortunately for us, the president took the long view.

Q: This was president Ollanta Moisés Humala Tasso.

A: Correct. I remember once saying to him that “for many of the things we are doing now, particularly all the infrastructure planning – building health centres and rural hospitals – you will not see the benefits”. Probably the next president will see some, and the next two presidents after him or her will get all the applause. And he said, “Okay, if that’s the way it’s going to be, go ahead.” I give him a lot of credit for that.

Q: I can see how being an economist might help in conversations with the finance minister, but what about doctors and hospital managers, or people developing health technologies?

A: As I said, I had been working with the health ministry for a long time, and so I had a good grasp of the basic issues, but it would be misleading to say that all of the conversations with the stakeholder groups were easy, including conversations with the providers of medical services and technology, and the health system’s bureaucrats. I am not singling out anyone for particular criticism, but making the broad point that because of established practices and different interests, not everyone is ready to embrace changes in the way things are done to benefit the population.

In many ways, ensuring that the focus of reform is on the needs of the population is the biggest challenge of all. When undertaking health system reform, you really need to keep in mind the impact of the changes you are implementing on the population.

